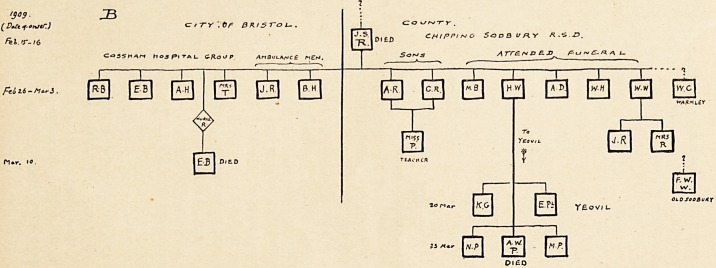# Small-Pox in Bristol and Neighbourhood (1908-9)

**Published:** 1909-06

**Authors:** D. S. Davies

**Affiliations:** Medical Officer of Health


The Bristol

Medico-Chirurgical Journal.

*"Scire est nescire, nisi id me
Scire alius sciret."*

JUNE, 1909.

### SMALL-POX IN BRISTOL AND NEIGHBOURHOOD (1908-9).

BY

D. S. Davies, M.D. Lond.,

*Medical Officer of Health.*

---

Three points lend interest to the introductions of small-pox which occupied the early months of 1909 :—

(1) The remarkable virulence and infectivity of the disease.

(2) The unusual circumstances accompanying the two main introductions of January and March.

(3) The manifestation of active hindrance to the Medical Executive arising from opposition to vaccination, and exerted, for the first time on record, through the Local Government of the city.

The first hint of forthcoming trouble was in December, 1908, when, after a year's freedom from small-pox, a corn-porter living in Bedminster, and at work within the incubation period on a steamship discharging grain at Portishead from Mariupol

(Sea of Azov), developed a mild attack of small-pox on December 14th, was removed to hospital, and discharged cured on January 23rd.

Before the first patient had left hospital, a man living in another part of the city sickened (about January 11th). In this instance, although no direct communication could be traced, the lapse of a double incubation period ($2 \times 14 = 28$ days) suggested causation through an intervening "missed" infection.

*The Tramcar Episode.*

This man unfortunately took the opportunity, before his illness was recognised and notified, of going by tramcar from the Horsefair, in the centre of the city, to Stapleton, on his way to the Workhouse, a ride of some three miles, traversing densely populated districts.

The control of small-pox notified immediately upon introduction into a family group in the city is fairly simple, involving merely the close observation and dealing with the comparatively few contacts at home or at work; and, by a judicious adjustment of revaccination, isolation, and other routine methods, has, over a series of years, proved effective. Thus in the ten years 1899–1908 the thirty-five introductions of the disease have resulted in a total of 136 cases, or an average yield per introduction of something under four cases.

But the circumstances under which this man became exposed—at the relieving offices, in the tramcar, and at work—involved the exposure to infection of 395 persons, who had to be kept under observation through a full incubation period, and this necessitated 5,147 visits of inquiry.

In addition to the known contacts, there must have been sundry others—passengers from and to places unknown—over whom no surveillance could be exercised. The problem was how to mark them down. Fortunately small-pox, even in its minimal forms, generally involves an initial fever of some severity, prompting resort to medical aid; so, as is usual, a warning notice was forwarded to every medical practitioner, briefly pointing out the facts, and noting the probable date of sickening. Notice was also sent to medical officers of health in adjacent districts.

This made no provision for those cases, the most dangerous of all, in which the initial symptoms, being slight, called only for domestic treatment, and in which the relief following a sparse eruption of papules would be followed by immediate resumption of work, in the confident belief that the illness was only a " touch of influenza cold," and that the ensuing eruption was evidence of its being successfully " driven out " by the remedies employed.

The contacts directly traceable to this case numbered four (see chart A). One other case in the same district was, in view of the time of sickening, probably a direct offshoot, and one of the Bedminster cases of January 29th may have been.

Of the directly resultant cases only one had issue, two men in a common lodging-house becoming infected, and these in turn infected a bootmaker friend, whose shop was a local gossip-house.

The two small circles shown in chart A under Mrs. D. and Mrs. R. represent two infants, unvaccinated at the time of their mother's attack, who were taken in, immediately vaccinated, and retained in the ward with their mother until discharge. The infants remained perfectly well.

It is possible also, as mentioned later, that an unknown passenger contracted the infection, and, himself an unrecognised (missed) case, went beyond the city and handed on the infection to the case in the country which started the Cossham outbreak. But this case may have had some other source.

*The Bedminster Group.*

The course of the disease in Bedminster has been spasmodic and disconcerting. While the outbreaks have been less obtrusive upon public attention, they have caused no less uneasiness to the medical department, for the disease has, rhizome-like, crept on beneath the surface, throwing up fatal buds at intervals.

The outbreak of January 5th, infecting one other member of a family, calls for no comment, though its origin was unknown.

The case of January 29th, possibly a contact of that of January 11th, infected only his daughter and a friend.

CITY OF BRISTOL.
SMALLPOX. DEC 1908 TO MAY 1909.
1908.
Dec 14
A.
A.M
(Dates of onset).
1909.
S. PHILIP GROUP.
BEDMINSTER GROUP.
M.G
Jan 5
Jan 11
E.D DIED
MRS G
Jan 16
Feb 2-3
MRS D
H.S
J.F
MRS R
MRS L
DIED
W.S
Jan 29
Feb 16
DIED A.H
F.C
NELLIE S
R.C
Feb 13
Feb 27
E.A D
LILY M.
Feb 21
H.A
Mar 8.
DIED BABY I.
Mar. 13
DIED E.A A
W.A.
V.A.
H.A
Mar 30
Ap. 3-4
MRS L.
MRS D.

1909.

(Date of onset.)

Feb. 15-16

Feb 26 - Mar 3.

Mar. 10.

B

CITY OF BRISTOL.

COSSHAM HOSPITAL GROUP.

AMBULANCE MEN.

R.B

E.B

A.H

MRS T

J.R

B.H

NURSE R.

E.B DIED

COUNTY.

CHIPPING SODBURY R.S.D.

?

J.S. R. DIED

SONS

ATTENDED FUNERAL

A.R.

C.R.

M.B

H.W

A.D.

W.H

W.W

W.C

WARMLEY

MISS P.

TEACHER

To YEOVIL

J.R

MRS R

F.W. W.

OLD SODBURY

20 Mar

K.G

E.Pl.

YEOVIL

23 Mar

N.P

A.W. P.

DIED

M.P.

On February 21st a disconnected mild case appeared in another part of Bedminster, and on March 13th a suddenly fatal case in an adjacent street.

On March 30th another suddenly fatal case led to the discovery of a "missed" infection of March 8th, of uncertain origin. Two of the members of this family, well vaccinated boys, V. A. and H. A., though exposed to intense infection, sickened, it is true, but their illness proceeded no farther than the initial fever, and in three days they regained perfect health without the sign of any eruption. Another unvaccinated son of 16 passed through a severe attack.

Again, in another part of the same district, a quickly fatal case on May 14th led to the discovery of another "missed" infection of mild type, the outcome of which has yet to be learnt.

*B. The Cossham Hospital Outbreak.*

The second invasion, or re-invasion, of the city was somewhat remarkable. A man, J. S. R., sickened about February 12th with *variola nigra*, at a village named Wick, in the Chipping Sodbury Rural District, Gloucestershire. The source of his infection was undetermined, but not improbably it was derived through a missed case—from E. D. during the tram-ride in Bristol on January 16th—as there are just a little over two incubation periods between January 16th and February 12th. The case was unfortunately mistaken for *purpura*, and conveyed by a private ambulance service on February 15th to the Cossham Hospital, a general hospital in Bristol. The patient died in the isolation ward on February 16th, a *post-mortem* was duly made, and the death returned as "acute nephritis." Hence no precautions were taken. The body was returned to Wick for burial.

Meanwhile, the sickening of two ambulance men in Bristol on February 27th led us to inquire what cases they had moved on or about February 15th (twelve days). The simultaneous appearance of the disease in the man's two sons, and in five other persons who carried the coffin or attended the funeral, soon made the matter clear.

Up to the morning of March 3rd, when we first arrived at a solution of the puzzle, the hospital authorities were still unaware of impending danger. The incubation period was overdue when we visited, so it was interesting to find that Mrs. T., a charwoman who cleaned out the isolation ward, and R. B., a clerk whose room adjoined the ward, were at home with "influenza." They were both removed to hospital in the papular stage of small-pox. Later a kitchenmaid and a wardmaid, who had been in the isolation block, also sickened, and were duly removed to hospital.

Towards the end of February two nurses, Sister W. and Nurse R., who had been revaccinated in 1908 had developed, we were told, a train of symptoms—fever, headache, etc., lasting two days—which might do duty either for "influenza" or for the "initial fever" of small-pox. No papules developed, they recovered perfectly, and Nurse R. immediately returned to duty in the ward on or about February 28th.

Twelve days afterwards, on March 10th, a patient in her ward developed small-pox, and was removed to hospital, where she died. She must have contracted the infection about February 28th.

It would appear, then, that with small-pox, as with typhoid fever, attendants on patients may, after slight indisposition, and without developing the characteristic symptoms of the disease, acquire temporary infectivity—become, in fact, "acute carriers." There is, fortunately, no reason to apprehend the discovery that a certain percentage of cases may become, upon apparent recovery, "chronic small-pox carriers," although the inclusion in a population of such continuously infective persons would do much to lessen the numbers of unvaccinated opponents to vaccination, unless due recognition of the fact had previously accelerated their conversion.

The city fared very well from this introduction. Not a single case occurred outside those immediately employed or resident at Cossham Hospital, and no infection was communicated to anyone after we took the matter in hand on March 3rd.

The county paid a somewhat heavier penalty. Seven cases received infection from the patient J. S. R., during his life or after his death. His two children had small-pox. The children's teacher suffered from a mild attack; one visitor to the funeral contracted the disease and carried it to Yeovil, where he infected five other persons, one fatally; another visitor suffered, and handed it on to two other inmates of his house. Two detached cases occurred also in the country—one at Cadbury Heath (Warmley), the other at Old Sodbury—but these proved sterile.

In all, this patient J. S. R. appears to have communicated infection, directly or indirectly, to twenty-five persons.

*The Ambulance Episode.*

The fact that a case of malignant small-pox had been conveyed in an ambulance used for general purposes naturally gave rise to some apprehension, which was not lessened by the fact of the sickening of both driver and attendant. It was found that thirty-eight cases had been removed in the same ambulance between February 16th and March 3rd to the various public hospitals, and to certain private residences in the city and neighbourhood. The ambulance management gave us free access to their books, from which we sorted out the cases, and despatched warning notices on the same evening, March 3rd, to the medical officers of the institutions concerned. It says much for the general care in cleanliness observed that, although no special measures of disinfection had been employed, as no suspicion of infection was entertained, not a single case of infection resulted to any one of the persons removed.

Personal infection is, of course, far more effective than that of "fomites," but the experiment, however fortunate the result in this instance, is not one to be repeated.

*Co-operation with the County.*

When the simultaneous infection of city and county occurred in March, I offered, with the consent of the Health Committee, to lend two experienced inspectors to assist in tracing out and watching contacts in the affected county areas. The plan worked very well, and the work of both inspectors has received deserved commendation. In the city, too, special inspectors were detailed for surveillance over the invaded and threatened city districts. They too worked thoroughly well.

*The Type of Disease.*

It must be admitted that the present introduction is of extremely virulent type. This is shown by the fact that the fatal case of May 14th is the third death from hemorrhagic small-pox, all in unvaccinated children, in the district of Bedminster alone, and by the further fact that out of the thirty-two city cases six have been hemorrhagic at onset or later. This gives the enormous percentage of 18.7 cases hemorrhagic in the present outbreak.

The infectivity is also intense. Practically every person who came into contact with the first case of the Cossham outbreak contracted the disease, and twenty-five cases are seen to have resulted directly or indirectly.

The intensity of infection is also suggested by the partial success of the disease in breaking down the very strong barrier of recent vaccination in the case of two nurses at Cossham and of the two boys in the Bedminster family; but in all four instances their acquired immunity bore the strain well, and, beyond the temporary inconvenience of a two days' fever, no harm of any kind resulted. It is in this way that these intense infections are apt to show a series of cases alternating from extremely mild attacks in the well-protected to malignant attacks in the unprotected.

*The Source of Introduction.*

The first case in December was, as stated, in a man who worked upon a steamship from foreign discharging grain. No other case of illness is known to have occurred on board.

This history is not an uncommon one. Thus in January, 1907, a first case of small-pox occurred in a man who worked as a labourer in warehousing ship-brought goods.

In April, 1891, a corn-porter working on foreign ships sickened of small-pox. No other case of illness was found upon the ship.

It is noteworthy also that the first case in the Cossham outbreak worked at a mill where part of the consignment from the Bristol ship was delivered, so that his infection may have been derived through infected sacks, rather than from secondary infection through a "missed" case from the tram-ride. It is also curious that the first family group in Bedminster, of January 5th, were relatives of the mill-owner; but the whole of the threads of causation are obscure, though suggestive of a distributed infection.

*The Work of Supervision.*

Some idea of the amount of work entailed by such outbreaks may be obtained from the number of contacts listed and visited between January 1st and May 1st. The contacts numbered 1,354, and the visits paid to them first and last were 16,398.

With an office staff and equipment designed only for work on a peace footing, this implies high pressure.

*The Cost of Precautionary Measures.*

The cost of the West Ham outbreak in 1902, which yielded 836 cases, was £29,381 17s. 4d.

The expenses of the present series of outbreaks have probably not yet amounted to £200 at a liberal estimate; but with so virulent a strain, and amongst a population still inadequately protected by vaccination and re-vaccination, the future of it is as yet on the knees of the gods.

Meanwhile, it is essential for ultimate success that the efforts of those directing the defensive measures should not only be unhampered, but actively supported.

*Opposition from the Anti-Vaccination Standpoint.*

The opposition to vaccination and to methods of control associated with vaccination, seeing whence it comes, can hardly be based on the merits or demerits of the case as a medical or scientific problem, but must have a deeper root in the mistaken idea that it has something to do with class legislation, and is an infringement of an Englishman's liberty to court infection. I confess that I greatly admire the courage of those unprotected persons who, never having seen small-pox, yet express a willingness to expose themselves to its infection. For my part I make no boast in the presence of malignant small-pox, which I have seen and studied, and continue to see and study, but which I should shrink from approaching were I not fully vaccinated, and did I not test my resistance by vaccination at least every year.

---

## Figures and Tables

**Figure f1:**
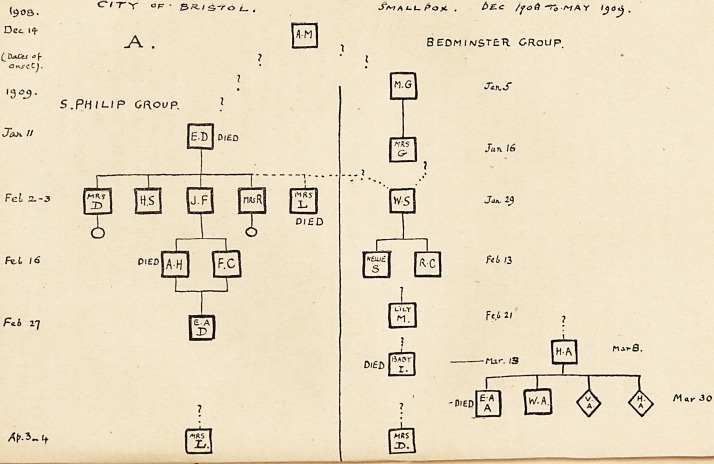


**Figure f2:**